# Pelvic Floor Muscle Training vs. Vaginal Vibration Cone Therapy for Postpartum Dyspareunia and Vaginal Laxity

**DOI:** 10.3390/medicina61010023

**Published:** 2024-12-27

**Authors:** Federico Villani, Izabella Petre, Florina Buleu, Stela Iurciuc, Luciana Marc, Adrian Apostol, Chiara Valentini, Elisabetta Donati, Tommaso Simoncini, Ion Petre, Cristian Furau

**Affiliations:** 1 Multidisciplinary Doctoral School, “Vasile Goldis” Western University of Arad, 310414 Arad, Romania; vllfrc@gmail.com; 2 Department of Obstetrics and Gynecology, “Victor Babes” University of Medicine and Pharmacy, 300041 Timisoara, Romania; 3 Cardiology Department, “Victor Babes” University of Medicine and Pharmacy, 300041 Timisoara, Romania; florina.buleu@umft.ro (F.B.); iurciuc.stela@umft.ro (S.I.); 4 Centre for Molecular Research in Nephrology and Vascular Disease, Faculty of Medicine, “Victor Babeș” University of Medicine and Pharmacy, 300041 Timisoara, Romania; marc.luciana@umft.ro (L.M.); apostol.adrian@umft.ro (A.A.); 5 Department of Internal Medicine II, Division of Nephrology, “Victor Babeș” University of Medicine and Pharmacy, 300041 Timisoara, Romania; 6 Department of Internal Medicine II, Division of Cardiology, “Victor Babeș” University of Medicine and Pharmacy Timisoara, 300041 Timisoara, Romania; 7 Department of Clinical and Experimental Medicine, UOC Obstetrics and Gynecology University I, University of Pisa, 56126 Pisa, Italy; valentinichiara08@gmail.com (C.V.); lisadonati@gmail.com (E.D.); 8 Department of Clinical and Experimental Medicine, Department of Surgical, Medical, Molecular and Critical Area Pathology, Department of Translational Research and New Technologies in Medicine and Surgery, University of Pisa, 56126 Pisa, Italy; tommaso.simoncini@med.unipi.it; 9 Doctoral School, “Victor Babes” University of Medicine and Pharmacy, 300041 Timisoara, Romania; petre.ion@umft.ro; 10 Department of Functional Sciences, Medical Informatics and Biostatistics Discipline, “Victor Babes” University of Medicine and Pharmacy, 300041 Timisoara, Romania; 11 Department of Pathophysiology, Faculty of Medicine, “Vasile Goldis” Western University of Arad, 310414 Arad, Romania; cristianfurau@gmail.com; 12 Department of Obstetrics and Gynecology, Emergency County Clinical Hospital of Arad, 310023 Arad, Romania

**Keywords:** pelvic floor dysfunction, postpartum, vaginal cones, pelvic floor muscle training, dyspareunia, sexual function

## Abstract

*Background and Objectives:* Pelvic floor dysfunction and sexual health issues are common postpartum due to weakened pelvic muscles, significantly impacting women’s quality of life (QoL). Pelvic floor muscle training (PFMT) is a widely used approach to address these issues. This study aimed to compare the effectiveness of two rehabilitation methods—vibrating vaginal cones (VCG) and PFMT exercises (CG)—in improving pelvic floor muscle strength, reducing dyspareunia, and enhancing sexual function in postpartum women. *Materials and Methods:* This 1-year retrospective observational analysis evaluated 57 postpartum women presenting with perineal muscle relaxation and sexual dysfunction. Participants were assessed 3 months postpartum (T0) and after 3 months of therapy (T1) at the Pelvic Floor Rehabilitation Clinic of Santa Chiara Hospital, Pisa. Outcomes were measured using the pubococcygeus (PC) test for pelvic floor strength and the Female Sexual Function Index (FSFI) for sexual function. *Results:* The results revealed significant improvements in pelvic floor muscle strength and sexual function across both groups. While both interventions effectively reduced dyspareunia, the VCG group demonstrated superior outcomes, with 96.67% of participants reporting no pain compared to 80.95% in the CG. FSFI scores improved significantly in both groups, with greater enhancements in arousal, desire, and pain domains observed in the VCG group (*p* < 0.01). Vaginal cone therapy also resulted in slightly higher gains in overall pelvic floor strength. *Conclusions:* These findings suggest that vibrating vaginal cones may be a promising option for postpartum pelvic floor rehabilitation, with potential benefits for improving sexual satisfaction and reducing pain.

## 1. Introduction

After delivery, women can present pelvic floor dysfunction either on a functional or a sexual scale. In fact, vaginal birth determines alterations to maternal connective tissue and perineal damage (childbirth lacerations) [1]. These conditions may promote the onset of other related conditions and adversely affect the quality of life (QoL).

One woman out of two reports having difficulties resuming sexual activity after delivery and presents pain during penetration [2,3].

After birth, numerous phenomena can affect sexual activity resumption: perineal tears and stitching can cause pain and discomfort; stretched muscle fibres and perineal tissues following delivery make the area sore and sensitive; weakness of the pelvic floor alters sexual perception and sensitivity and, therefore, reduces satisfaction [4]; and breastfeeding can reduce libido (reduction of testosterone and raised prolactin).

The most frequent postpartum sexual dysfunctions are discomfort/pain during sexual activity (dyspareunia), loss of desire, lower levels of sexual pleasure and emotional satisfaction, and difficulty in reaching orgasm. To improve sexual performance and quality of life (QoL), reducing the risk factors associated with perineal dysfunction is essential [5,6]. Pelvic floor rehabilitation encompasses a range of non-surgical and non-pharmacological techniques designed to prevent or treat perineal dysfunctions. It is particularly effective in managing and preventing urinary incontinence and can be enhanced by combining it with other complementary methods [7].

One of the primary rehabilitation techniques is Pelvic Floor Muscle Training (PFMT), also known as Kinetic PreProcessor (KPP), which focuses on improving pelvic floor muscle function through targeted contraction and relaxation exercises [8]. Other methods include Functional Electrical Stimulation (FES), which provides passive muscular stimulation using superficial pads or vaginal/rectal probes that deliver low-energy electrical pulses. Biofeedback (BFB) enables individuals to gain better control over involuntary bodily processes through visual, tactile, and auditory feedback. Vaginal devices, such as spheres or cones, are also employed in perineal rehabilitation by using feedback from voluntary and involuntary muscle contractions [9,10,11], often in conjunction with gravity, and can be used individually or with increasing weights for progressive strengthening.

This study aims to assess the comparative efficiency of innovative vibrating vaginal cone therapy versus conventional pelvic floor muscle exercises in addressing postpartum sexual dysfunctions and pelvic muscle relaxation. By comparing the effectiveness of these interventions, the study seeks to inform clinical practices and offer evidence-based recommendations for postpartum care.

## 2. Materials and Methods

### 2.1. Study Design and Participants

This retrospective observational study assessed the outcomes of a randomized comparative study conducted at the Pelvic Floor Rehabilitation Clinic of Santa Chiara Hospital, Pisa, between November 2017 and November 2018. The study enrolled 70 postpartum women with perineal muscle relaxation and sexual dysfunction 3 months after delivery. Participants were randomly assigned to either the vaginal cone group (VCG, n = 35) or the pelvic floor muscle training group (CG, n = 35). A total of 57 participants completed the program, with 32 in the VCG and 25 in the CG.

The study included postpartum women with perineal muscle relaxation or sexual dysfunction and without contraindications for pelvic floor rehabilitation. Exclusion criteria comprised ongoing pregnancy, unresolved vaginal epithelial healing, active infections, vaginal mucosal disorders, or vulvovaginal pain requiring medical or alternative interventions.

Participants were assessed at baseline (T0, 3 months postpartum) and after 3 months of therapy (T1, 6 months postpartum). At T0, medical and obstetric histories were collected, including age, body mass index (BMI), delivery mode, perineal tears, and reports of dyspareunia or urinary incontinence. Pelvic floor muscle strength was evaluated using the pubococcygeus test, which assessed phasic force, endurance, and fatigue. Sexual function was measured using the Female Sexual Function Index (FSFI), a questionnaire covering six domains: desire, arousal, lubrication, orgasm, satisfaction, and pain [12]. Dyspareunia severity was self-reported and classified as superficial pain (S), occurring at the vestibular or introital level; moderate pain (SP), originating in the vaginal compartment; or profound pain (P), associated with the cervical or uterine area.

### 2.2. Intervention Protocol

The VCG used a single vibrating vaginal cone made from medical-grade silicone, which reduces the risk of irritation or injury. It weighed 37 g and had a smooth, seamless design, making it easier to insert and remove without causing abrasions. Participants were instructed to insert the cone about 3 cm into the vaginal canal, using a water-based lubricant to ensure comfort and reduce friction. Its design leveraged gravity to stimulate involuntary muscle contractions, while an internal steel ball generated vibration, providing biofeedback and enhancing neuromuscular coordination. Participants used the cone for 1 h daily during light physical activities, promoting targeted pelvic floor engagement and improved muscle function. The use of the cone was periodically supervised to ensure proper technique and adherence to the protocol, helping to maintain the safety and effectiveness of the intervention.

The CG performed traditional Kegel exercises, focusing on isolating the pelvic floor muscles while avoiding engagement of the abdominal or gluteal muscles. The daily regimen included 50 maximal voluntary contractions and 20 prolonged submaximal contractions. Exercises were initially performed in a modified gynecological position, progressing to sitting and standing postures to increase complexity. Both groups received individualized guidance to ensure adherence and proper technique throughout the rehabilitation program.

### 2.3. Assessment Methods

Pelvic floor muscle strength was evaluated using the pubococcygeus test, which measured three parameters: phasic force (strength of a single contraction), endurance (duration of sustained contraction), and fatigue (capacity to perform repeated contractions without exhaustion) [13]. Each parameter was scored on a standardized scale:

- Phasic force (FF): refers to the maximal, rapid contraction lasting 1 s and is graded using a scale with 5 grades: 0 (no contraction), 1 (very weak contraction, felt as a slight shiver), 2 (weak but distinct contraction), 3 (moderate contraction without resistance), 4 (strong contraction with moderate resistance), and 5 (maximal contraction with strong resistance).
- Endurance (E): measures how long the maximal contraction can be maintained, scored from 0 to 3: 0 (less than 2 s), 1 (2 to 5 s), 2 (5 to 9 s), and 3 (more than 9 s).
- Fatigue (F) was assessed through 10 maximal contractions, each lasting 5 s with 10 s rest intervals, and is graded from 0 to 3 based on the number of completed contractions: 0 (fewer than 2 contractions), 1 (2 to 5 contractions), 2 (5 to 9 contractions), and 3 (more than 9 contractions).

Sexual function was assessed using the FSFI, a validated questionnaire that evaluated six domains: desire, arousal, lubrication, orgasm, satisfaction, and pain. Each domain was scored from 0 to 6, with higher scores indicating better function. For the pain domain specifically, a score of 6 means no pain during sexual activity, reflecting the best possible outcome. The total FSFI score ranges from 2 to 36, offering a detailed way to assess overall sexual health.

Participants were asked to complete the FSFI questionnaire at least 48 h after their last sexual activity to avoid their most recent experiences influencing their answers (a concept known as recency bias). They were also instructed to base their responses on their average sexual experiences over the past 4 weeks, which is the standard guideline for using the FSFI [12]. This helped reduce inaccuracies caused by memory issues (recall bias).

To ensure consistency, the FSFI was administered under the same conditions at two points during the study: at the beginning (T0, 3 months postpartum) and after the intervention (T1, 6 months postpartum). This method allowed us to directly link any improvements in sexual function to the therapies used in the study.

### 2.4. Statistical Analysis

Data were collected with Microsoft Excel 2021 program and analyzed descriptively and analytically with JASP v18.3. The Shapiro–Wilk test was used to assess data distribution, which showed that the data were not normally distributed, requiring the use of non-parametric tests. For continuous variables, mean, median, and standard deviation (SD) were calculated, while percentages and frequencies summarized qualitative data. To compare groups at the same time points, the Mann–Whitney test was applied for continuous variables, and the chi-square test was applied for categorical variables. To examine changes over time in the PC test and FSFI scores, the Wilcoxon Signed Rank test, a non-parametric equivalent of the paired samples T-test, was used. A significance level of α = 0.05 was set for determining statistical significance, and effect sizes (ES) were calculated to measure the magnitude of changes in the parameters, with effect sizes interpreted as small (0.1–0.2), moderate (0.3–0.4), and large (>0.5) [14].

## 3. Results

Of an initial sample of 70 women, 9% of VCG and 29% of CG dropped out due to motivation and time constraints. As a result, the statistical analysis was performed on a global sample of 57 women divided into two groups, 32 of them using vaginal cones (VCG) and 25 practicing PFMT (CG).

There are no significant differences (*p* > 0.05) in age, BMI, parity, episiotomy rates, or laceration grades between the two groups. Superficial (S) and severe (SP) dyspareunia were more common in the VCG at baseline (*p* = 0.017). Pelvic floor muscle strength was comparable between groups, indicating similar initial conditions in terms of pelvic floor function. The FSFI score, an indicator of sexual function, was significantly higher in the VCG, implying better sexual function at baseline compared to the control group (*p* = 0.030) (Table 1).

**Figure 1:**
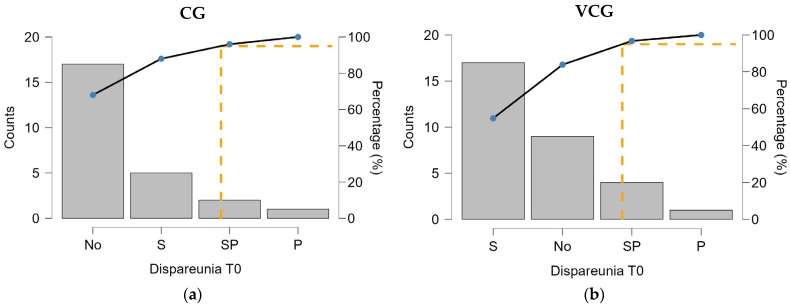
Distribution of dyspareunia by groups, at T0: (**a**)-CG, (**b**)-VCG.

At baseline (T0), CG had a higher proportion of participants reporting no dyspareunia (68%) compared to VCG (31.25%), and fewer participants with superficial (S) (20% vs. 53.12%) or moderate (SP) pain (8% vs. 12.5%). Both groups had a small number of participants with profound dyspareunia (P) (4% in CG and 3.12% in VCG). The chi-squared test revealed a statistically significant difference in the distribution of dyspareunia between the two groups (*p* = 0.014), indicating that dyspareunia was more frequent in the VCG at T0 (Figure 1).

**Figure 2:**
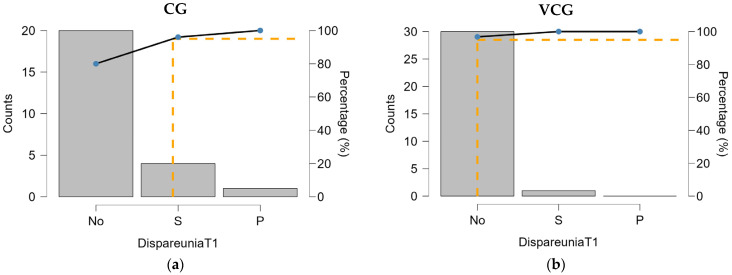
Distribution of dyspareunia by groups, at T1: (**a**)-CG, (**b**)-VCG.

After therapy, both groups experienced significant improvements in dyspareunia, with the vast majority of women reporting no pain. In the vaginal cone group (VCG), 96.67% of participants reported no dyspareunia, compared to 80.95% in the control group (CG). Although both groups improved, the reduction in dyspareunia was more pronounced in the VCG, where only one woman (3.33%) still reported superficial pain, compared to four women (19.05%) in the CG (Figure 2). The chi-squared test at T1 showed that the differences between the two groups were no longer statistically significant (*p* = 0.063). This indicates that both interventions were effective in reducing dyspareunia, but the vaginal cones may offer a slight edge in enhancing pain relief.

Both the control group (CG) and vaginal cone group (VCG) showed significant improvements in pelvic floor muscle function, as measured by the PC test. In the CG, phasic force, endurance, and fatigability all increased significantly from T0 to T1 (*p* < 0.001), with small-to-moderate effect sizes (ES ranging from 0.225 to 0.250). Similarly, the VCG demonstrated significant improvements across all metrics (*p* < 0.001), with comparable effect sizes (ES ranging from 0.203 to 0.229). The total PC score improved significantly in both groups, increasing from 2.740 to 5.360 in the CG and from 2.661 to 5.694 in the VCG. While both groups experienced substantial gains in pelvic floor muscle function, the VCG showed slightly higher overall total scores at T1, suggesting a marginal advantage with the vaginal cone intervention. However, the effect sizes indicate that the magnitude of improvement was similar in both groups (Table 2).

**Figure 3:**
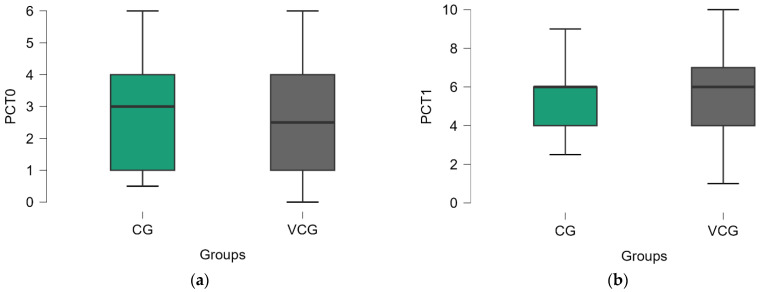
PC test score distribution by groups: (**a**) at T0, (**b**) at T1.

The results from the Mann–Whitney U Test (used here for comparing the two groups at baseline and after therapy) revealed no significant differences between the two groups at baseline (T0) or after therapy (T1) for any of the evaluated parameters (*p* > 0.05). This suggests that while both interventions were effective in improving pelvic floor muscle function, neither group showed a statistically superior outcome over the other at either time point (Table 3 and Figure 3).

At the level of sexual function, both groups showed statistically significant improvements across all FSFI domains post-therapy. In the CG, arousal, desire, orgasm, satisfaction, pain, and lubrication scores all increased significantly (*p* < 0.01), with effect sizes (ES) ranging from 0.225 to 0.328. The total FSFI score for CG increased from 19.488 to 23.428 (*p* < 0.001), showing a moderate overall improvement in sexual function. Similarly, the VCG demonstrated significant improvements across all domains (*p* < 0.001), with slightly higher effect sizes compared to the CG, ranging from 0.206 to 0.262. The total FSFI score in the VCG rose from 23.332 to 29.235 (*p* < 0.001), indicating a more pronounced improvement in sexual function than in the CG (Table 4). These results suggest that while both groups experienced substantial gains, the VCG showed greater overall enhancement in sexual function.

The comparison between the control group (CG) and the vaginal cone group (VCG) showed that after therapy (T1), both groups improved significantly, but the VCG consistently achieved higher mean scores in each domain, notably in arousal (4.732 vs. 3.744, *p* = 0.004), desire (4.394 vs. 3.264, *p* = 0.001), and pain (5.355 vs. 4.640, *p* = 0.004). The total FSFI score improved significantly in both groups, but the VCG showed a greater increase (from 23.332 to 29.235, *p* < 0.001) compared to the CG (from 19.488 to 23.428, *p* < 0.001). These results suggest that while both interventions effectively enhanced sexual function, the vaginal cone therapy led to superior outcomes across all domains (Table 5).

**Figure 4:**
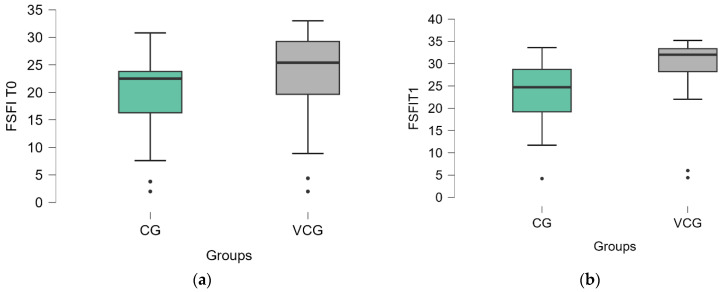
FSFI score distribution by groups: (**a**) at T0, (**b**) at T1.

The evolution of FSFI within the two groups is illustrated in Figure 4.

Although statistically significant improvements were observed in FSFI scores and pelvic floor muscle strength in both groups, it is important to note that the changes, particularly in the VCG group, translate to meaningful reductions in dyspareunia and enhancements in sexual satisfaction. The high percentage of women reporting no pain post-therapy in the VCG (96.67%) and the substantial improvement in satisfaction and arousal scores highlight the practical benefits of this intervention for postpartum women. These improvements may have a significant impact on quality of life, as sexual function and pain relief are key concerns during the postpartum period.

## 4. Discussion

Although various studies have examined the role of vaginal cones in treating pelvic floor dysfunction (PFD), conclusions about their effectiveness compared to other conservative treatments remain controversial [15]. This study evaluated the comparative effectiveness of traditional PFMT with simple exercises and vibrating vaginal cones (VC) in treating postpartum vaginal relaxation, dyspareunia, and sexual dysfunction. However, the study is one of the few to specifically address the impact of vaginal cones on sexual function, an area where research is still limited.

In our study, all participants had vaginal deliveries, but we observed notable differences in baseline results between the two groups. The severity of perineal tears appeared to influence both baseline dyspareunia and sexual function outcomes. As shown in Table 1, the VCG had a higher proportion of women with second- and third-degree tears (31.25% and 9.38%, respectively) compared to the CG (20.0% and 0%). This greater level of perineal trauma likely contributed to the higher prevalence of dyspareunia in the VCG at baseline despite their higher FSFI scores. After the intervention, both groups showed significant improvements. However, women in the VCG seemed to benefit more, particularly in terms of pain reduction and improved sexual satisfaction, even for those with severe dyspareunia at the start. This suggests that the targeted stimulation and biofeedback provided by the vibrating vaginal cones may help overcome the challenges associated with more severe perineal trauma, leading to more effective recovery.

The device used in this study is the Pelvik single vaginal cone suitable for postpartum perineal laxity and weakness. The cone is designed in a new and physiological shape and made of medical silicone. The firm body and flexible tail are made of the same material without connections, incisions, or gluing. Because of this, it is possible to avoid abrasions during its application/removal and lower the risk of infections. Inside the cone, there is a steel ball that runs freely during the exercise, hitting the prismatic cavity walls and generating vibrations. These enhance muscular biofeedback and efficiency more efficiently than passive muscle training. Once inserted in the vaginal canal, Pelvik, due to its physiological shape, descends with gravity and causes voluntary contractions of perineal muscles.

The biofeedback mechanism and the resistance provided by the cones may enhance muscle activation and coordination, leading to more rapid and targeted improvements [16]. Vaginal cones are often used as part of pelvic floor muscle training (PFMT), which has been associated with improvements in sexual function, particularly in areas such as arousal, orgasm, and satisfaction. Research shows that strengthening the pelvic floor muscles can enhance sexual sensation and orgasm, as well as boost confidence and sexual satisfaction [16,17]. Previous research has demonstrated the effectiveness of pelvic floor muscle training in evaluating both pelvic floor muscles and female sexual function [18,19].

Our study showed significant reductions in dyspareunia for both groups following therapy. At baseline, dyspareunia was more prevalent in the VCG group, where only 31.25% of participants reported being pain-free, compared to 68% in the CG. After the intervention, there was a notable improvement in both groups, with 96.67% of participants in the VCG achieving complete pain relief compared to 80.95% in the CG (Figure 2). Furthermore, the number of women experiencing superficial pain reduced substantially in the VCG (3.33%) compared to the CG (19.05%). The chi-squared test at T1 showed that the difference between the two groups was no longer statistically significant (*p* = 0.063). This suggests that both interventions were highly effective in reducing dyspareunia, but the vaginal cone therapy may offer an additional benefit in accelerating the reduction of pain. These results support the potential advantage of early intervention with vaginal cones in improving pelvic floor function and reducing dyspareunia.

Women experiencing dyspareunia report pain during intercourse, which is frequently caused by trigger points in the PFMs. Studies suggest that transvaginal approaches, such as myofascial release techniques and massage, enhance blood flow to the PFMs and help release muscle tension and trigger points, thereby interrupting the cycle of genito-pelvic pain and PFM overactivity [20,21]. Additionally, digital biofeedback and patient education, as part of a comprehensive treatment plan, play key roles in alleviating sexual pain and reducing muscle overactivity [22]. Improving muscle awareness, enhancing proprioception, promoting muscle relaxation, and restoring normal PFM function contribute to reducing pain associated with sexual pain disorders [23].

The PC test results indicated significant improvements in pelvic floor strength, endurance, and fatigability for both groups (*p* < 0.01) (Table 2), which aligns with previous research on PFMT’s efficacy in improving pelvic floor muscle function [24,25]. The improvements were comparable between the two groups as evidenced by the Mann–Whitney U Test, which showed no significant differences between the VCG and CG in pelvic floor parameters at both the baseline and after therapy (Table 3 and Figure 3a,b).

As shown in Table 4, both groups showed improvements across all FSFI domains, with the VCG demonstrating significantly greater gains, particularly in the satisfaction and pain domains (*p* = 0.007 and *p* = 0.004, respectively) (Table 5). These findings are consistent with other studies that have highlighted the effectiveness of vaginal cones in enhancing sexual function and comfort by targeting pelvic floor muscle strength and neuromuscular control [18,19].

While both interventions significantly improved pelvic floor muscle strength and sexual function, it is important to consider how these changes translate into clinical benefits. The reduction in dyspareunia (pain during intercourse) was particularly notable in the VCG group, where 96.67% of participants reported being pain-free after the intervention compared to 80.95% in the control group. This suggests that vibrating vaginal cones may offer meaningful relief for postpartum women dealing with sexual pain. Additionally, improvements in FSFI domains—such as satisfaction, arousal, and lubrication—observed in the VCG group are likely to enhance overall sexual health and emotional well-being, both of which are vital for postpartum quality of life.

Although the difference in pelvic floor muscle strength (PC test) between the two groups was not statistically significant, the comparable improvements in both interventions underscore the importance of pelvic floor rehabilitation programs in addressing postpartum dysfunction. These findings highlight the need for therapies that not only restore pelvic floor function but also reduce symptoms like dyspareunia and improve quality of life.

The targeted stimulation and biofeedback provided by the vibrating cones likely contributed to faster pain relief and better sexual satisfaction, offering a distinct advantage over simple pelvic floor exercises. However, we acknowledge certain limitations of this study. The small sample size and short follow-up period may limit the generalizability of our findings. Additionally, the higher dropout rate in the control group could have influenced the comparison between interventions. While the improvements observed were statistically significant, future studies are needed to confirm whether these changes have a lasting impact on women’s daily lives. The retrospective nature of the study is another limitation, as it restricts control over certain variables and may affect the reliability of our conclusions. Furthermore, we recognize a potential conflict of interest since one of the authors is the inventor of the vaginal cone. To mitigate bias, the inventor was excluded from participant recruitment, data collection, and analysis. Nonetheless, this should be considered when interpreting the results.

To strengthen these findings, future research should include larger, more diverse samples, extend follow-up periods, and adopt rigorous study designs, such as randomized controlled trials. Additionally, exploring the psychological impact of these therapies, patient satisfaction, and their influence on intimate relationships would provide a more comprehensive understanding of how vibrating vaginal cones compare to other rehabilitation options.

## 5. Conclusions

Both vibrating vaginal cones and pelvic floor muscle training (PFMT) were effective in improving pelvic floor muscle strength, reducing dyspareunia, and enhancing sexual function in postpartum women. The vibrating vaginal cones showed some advantages, particularly in pain relief and sexual satisfaction, which may be attributed to their biofeedback and resistance mechanisms.

While promising, these results should be interpreted with caution due to the small sample size and short follow-up period of this study. Further research with larger sample sizes, diverse populations, and longer follow-up periods is necessary to validate these findings and assess their long-term effectiveness.

Vibrating vaginal cones could be considered a complementary option in postpartum rehabilitation programs, particularly for women experiencing pelvic floor dysfunction and sexual discomfort. However, additional evidence is needed before widespread implementation in clinical practice.

## Figures and Tables

**Table 1:** Baseline characteristics.

Demographic and Clinic Parameters	CG	VCG	*p*
Years (mean ± SD)	34.60 ± 5.55	36.656 ± 4.077	0.232
BMI—Kg/m^2^ (mean ± SD)	22.60 ± 2.49	23.31 ± 4.25	0.917
Number of vagina delivery (N/%)			0.577
1	15/60	23/71.88	
2	8/32.0	6/18.75	
3	2/8.0	3/9.38	
Episiotomy (N/%)			0.798
No	11/44.0	13/40.63	
Yes	14/56.0	19/59.38	
Laceration grade (N/%)			0.237
0	13/52.0	14/43.75	
1	7/28.0	5/15.63	
2	5/20.0	10/31.25	
3	0/0.0	3/9.38	
Dyspareunia T0			0.017
No	17/68.0	10/31.25	
p	1/4.0	1/3.12	
S	5/20.0	17/53.12	
SP	2/8.0	4/12.5	
PC test score (mean ± SD)	2.74 ± 1.62	2.70 ± 1.63	0.895
FSFI T0	19.49 ± 7.94	23.33 ± 7.960	0.030

**Table 2:** PC test score evolution in each group.

PC Test Score	T0	T1	*p* *	ES
**CG**				
Phasic force	1.340 ± 0.515	2.440 ± 0.870	<0.001	0.250
Endurance	0.680 ± 0.627	1.620 ± 0.754	<0.001	0.244
Fatigability	0.720 ± 0.614	1.700 ± 0.736	<0.001	0.239
PC total score	2.740 ± 1.615	5.360 ± 1.929	<0.001	0.225
**VCG**				
Phasic force	1.597 ± 0.638	2.661 ± 0.943	<0.001	0.225
Endurance	0.532 ± 0.645	1.484 ± 0.758	<0.001	0.229
Fatigability	0.532 ± 0.591	1.565 ± 0.772	<0.001	0.221
PC total score	2.661 ± 1.640	5.694 ± 2.088	<0.001	0.203

* Wilcoxon saint rank test.

**Table 3:** PC test score evolution by groups.

PC Test	Group	T0	T1
		*p* *	*p* *
Phasic force	CG	0.090	0.325
	VCG		
Endurance	CG	0.364	0.497
	VCG		
Fatigability	CG	0.269	0.500
	VCG		
Total	CG	0.986	0.407
	VCG		

* Mann–Whitney U test.

**Table 4:** FSFI score evolution in each group.

FSFI Domains	T0	T1	Df	*p* *	ES
**CG**					
Arousal	3.084 ± 1.644	3.74 ± 1.535	−3.571	<0.001	0.262
Desire	2.904 ± 1.158	3.264 ± 1.250	−2.578	0.010	0.328
Orgasm	2.848 ± 1.779	3.536 ± 1.611	−3.296	0.001	0.294
Satisfaction	3.488 ± 1.388	4.144 ± 1.418	−2.746	0.006	0.269
Pain	3.504 ± 2.243	4.640 ± 1.523	−3.296	0.001	0.294
Lubrication	3.660 ± 1.992	4.320 ± 1.544	−3.284	<0.001	0.277
Total	19.488 ± 7.943	23.428 ± 7.034	−4.319	<0.001	0.225
**VCG**					
Arousal	3.939 ± 1.596	4.73 ± 1.491	−3.920	<0.001	0.250
Desire	3.406 ± 1.281	4.394 ± 0.935	−4.197	<0.001	0.234
Orgasm	4.245 ± 1.616	4.839 ± 1.574	−3.148	0.002	0.234
Satisfaction	3.819 ± 1.619	5.058 ± 1.313	−4.171	<0.001	0.229
Pain	3.897 ± 1.981	5.355 ± 1.305	−4.107	<0.001	0.239
Lubrication	4.026 ± 1.902	4.887 ± 1.567	−3.724	<0.001	0.262
Total	23.332 ± 7.960	29.235 ± 7.260	−4.782	<0.001	0.206

* Wilcoxon saint rank test.

**Table 5:** FSFI score evolution by groups.

FSFI Domains	Group	T0	T1
		*p* *	*p* *
Arousal (A)	CG	0.049	0.004
	VCG		
Desire (D)	CG	0.127	0.001
	VCG		
Lubrication (L)	CG	0.451	0.051
	VCG		
Orgasm (O)	CG	0.002	<0.001
	VCG		
Satisfaction (S)	CG	0.333	0.007
	VCG		
Pain (P)	CG	0.502	0.004
	VCG		
FSFIT	CG	0.030	<0.001
	VCG		

* Mann–Whitney U test.

## Data Availability

The data presented in this study are available on request from the corresponding author. The data are not publicly available due to privacy reasons.
